# Surface/Interface Engineering for Constructing Advanced Nanostructured Light-Emitting Diodes with Improved Performance: A Brief Review

**DOI:** 10.3390/mi10120821

**Published:** 2019-11-27

**Authors:** Lianzhen Cao, Xia Liu, Zhen Guo, Lianqun Zhou

**Affiliations:** 1 Department of Physics and Optoelectronic Engineering, Weifang University, Weifang 261061, China; lianzhencao@wfu.edu.cn; 2 CASKey Lab of Bio-Medical Diagnostics, Suzhou Institute of Biomedical Engineering and Technology, Chinese Academy of Sciences, Suzhou 215163, China; 3 Shandong Guo Ke Medical Technology Development Co., Ltd., Jinan 25001, China; 4 Zhongke Mass Spectrometry (Tianjin) Medical Technology Co., Ltd. Tianjin 300399, China; 5 Jihua Laboratory, Foshan 528200, China

**Keywords:** nanostructured materials, surface/interface properties, nanostructured light-emitting devices, physical mechanism, surface/interface modification, surface/interface control

## Abstract

With the rise of nanoscience and nanotechnologies, especially the continuous deepening of research on low-dimensional materials and structures, various kinds of light-emitting devices based on nanometer-structured materials are gradually becoming the natural candidates for the next generation of advanced optoelectronic devices with improved performance through engineering their interface/surface properties. As dimensions of light-emitting devices are scaled down to the nanoscale, the plentitude of their surface/interface properties is one of the key factors for their dominating device performance. In this paper, firstly, the generation, classification, and influence of surface/interface states on nanometer optical devices will be given theoretically. Secondly, the relationship between the surface/interface properties and light-emitting diode device performance will be investigated, and the related physical mechanisms will be revealed by introducing classic examples. Especially, how to improve the performance of light-emitting diodes by using factors such as the surface/interface purification, quantum dots (QDs)-emitting layer, surface ligands, optimization of device architecture, and so on will be summarized. Finally, we explore the main influencing actors of research breakthroughs related to the surface/interface properties on the current and future applications for nanostructured light-emitting devices.

## 1. Introduction

The area of nanostructure materials refers to a new system that is composed or assembled according to certain rules based on the material units at the nanoscale (usually below 100 nm). According to the spatial dimension of the nanometer scale, nanostructured materials can be divided into zero-dimensional (0D) nanometer materials (such as nanoparticles, artificial atoms, clusters, etc.), one-dimensional (1D) nanomaterials (such as nanowires, fine rice filaments, nanorods, nanotubes, and nanofibers, etc.), and two-dimensional (2D) nanomaterials (such as nanoribbons, nanoscale disks, superlattices, multilayer membranes, etc.). As nanomaterials are in line with development trends related to the miniaturization and miniaturization of future devices, they will be regarded as the basic component unit of various components in the future once discovered; furthermore, they have been widely considered already [1,2,3,4,5,6,7,8,9,10].

In addition, the quantum confinement, size, and surface/interace effects of nanometer materials make them different from similar component bulk materials in mechanics, calorifics, optics, electricity, magnetism, and so on. Many of the physical properties such as optical properties can be controlled and optimized through the appropriate method. So, nanomaterials in both science and engineering fields have been widely studied and used. In the early research on nanomaterials, people usually started from the synthesis of nanomaterials and studied the changes of material properties by adjusting the composition and structure of materials. At the same time, the continuous development of micro/nanoprocessing technology promotes the research of nanodevices based on nanomaterials and helps people to better find the relationship between material properties and device performance, as well as deeply understand the novel physical phenomena in the nanometer world.

Nowadays, photoelectric devices, which can be broadly divided into three categories, play an indispensable role in people’s daily lives. First, devices are used as light sources to convert incoming electrical energy into light radiation, such as light-emitting diodes (LEDs) and laser diodes (LDs). The second is an optical detector, which converts weak light signals into the electrical signals of devices such as photomultiplier tubes, photodiodes, and phototransistors. The third category is photovoltaic devices, or solar cells, which convert light into electricity. From the classification of photoelectric devices, we can see that the interaction between photons and electrons in materials plays an important role in the process of energy conversion from electrons to photons or photons to electrons, and is an important determinant of device performance. Compared with the traditional optoelectronic devices with bulk materials, nanostructured optoelectronic devices, such as LEDs, have attracted more and more attention due to their high quantum efficiency, flexibility, and stability [11,12,13,14,15], and some nanostructure LEDs have been applied commercially. For example, the industrialization of organic light-emitting diode (OLED) technology has been realized, and it is gradually applied in the commercial field, such as mobile phones, digital cameras, and televisions [11,12,13,14,15].

Similar to traditional light-emitting devices, the energy conversion between photons and electrons or between electrons and photons of nanostructured light-emitting devices usually involves the generation of carriers, recombination, separation, collection, and other dynamic processes, all of which are closely related to the surface/interface of devices. When the size of the device is scaled down to the nanometer level, the proportion of the surface/interface in the device increases sharply. Thus, the surface/interface properties of nanostructured materials are an important factor affecting the performance of the photoelectric devices [16,17,18,19,20]. So, how to explore the surface/interface properties of nanostructured light-emitting devices for constructing advanced light-emitting devices with improved performance has become a hot pursuit for the researchers. We need to comprehensively study the surface/interface of nanostructured light-emitting devices from the microscopic perspective in depth and understand the correlation between the properties of nanostructured devices and the physical mechanism of the surface/interface in the materials and devices. Here, in this paper, how the surface/interface properties dominating the performance of the built photoelectric devices will be illustrated theoretically and summarized experimentally.

## 2. Surface/Interface Properties

**Figure 1:**
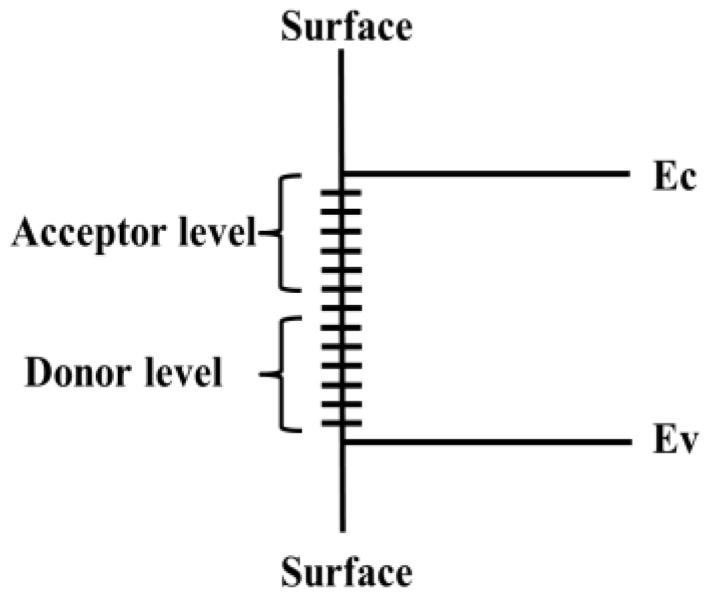
Schematic diagram of surface level.

The surface is broadly defined as all the atomic layers that do not have the three-dimensional periodicity of bulk materials. The presence of the nanosurface will destroy the periodicity of the three-dimensional infinite lattice in the direction of the vertical surface. Therefore, the potential energy of the lattice electrons does not have translational symmetry in the direction of the vertical surface. If the Schrodinger equation is solved directly by using the theory of quantum mechanics, some new eigenvalues will appear in the Hamiltonian eigenvalue spectrum. This means that there will be a new electronic energy state (energy level), which is defined as a surface state, as shown in Figure 1. There are two kinds of surface states; one is called an intrinsic surface state, which is the electronic state on the surface without foreign impurities. The other is called the induced surface state, which is due to the presence of impurities, adsorbed atoms, and other imperfections on the surface. According to the different action with the electron, the surface state also can be divided into a donor state and acceptor state. The donor state is a neutral surface state occupied by an electron. A negatively charged surface state is occupied by an electron. Surface states have three very important characteristics: first, they can be the effective generation and composite center of a few carriers in the material, which determines the surface composite characteristics; second, the surface state scatters most carriers, reduces the surface mobility, and affects the surface conductance; third, the electric field generated on the vertical material surface causes the surface electric field effect [21,22,23].

The interface refers to the interface between different substance phases or different kinds of substances. Similar to surface states, interface states are introduced at the interface state and occur at the interface where two different substances are in contact or at the interface of a heterogeneous junction due to the interruption of a periodic lattice constant. These interface states can also be introduced by lattice mismatch and interface roughness. In addition, the interface state can also be generated because the thermal expansion of the two materials does not match. The interface state is a local electronic state that cannot propagate in the material. Interface states are generally divided into a donor and recipient. Regardless of the position of the energy level in the forbidden band, if the energy level is neutral when occupied by electrons and positively charged after releasing electrons, it is called the donor interface state. If the energy level is neutral when it is empty and the electron is negatively charged, it is called the acceptor interface state. The interface state is another key factor to determine the performance of nanostructured devices [24,25,26].

**Figure 2:**
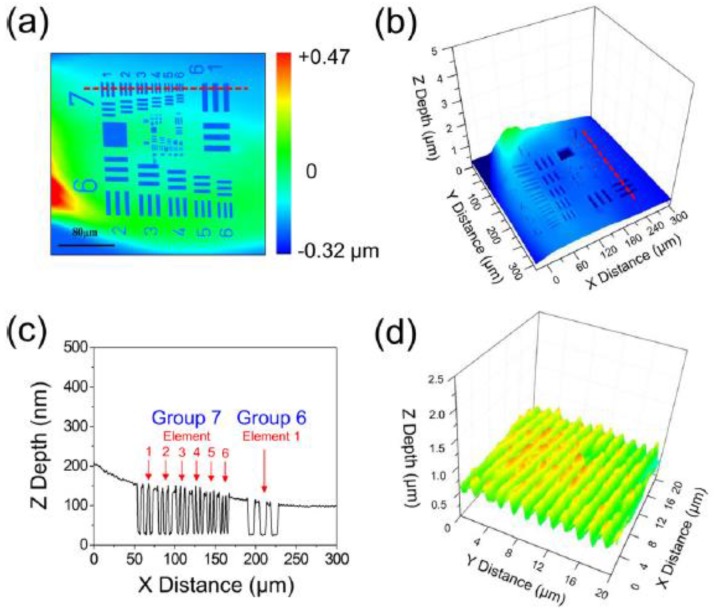
Three-dimensional (3D) surface profiles of metallic film. (**a**) Top view. (**b**) Perspective view. (**c**) Cross-sectional view. (**d**) Perspective view of a 600 line/mm diffraction grating. Reproduced with permission from reference [27].

As mentioned above, theoretically studying the influence of surface interface on nanomaterials and devices is of great significance to improve the performance of nanostructured devices. Fortunately, with the development of material characterization technology, there are many means and instruments to characterize the properties of surface interfaces, which can be used to validate and promote theories and help scientists to understand their impact on nanomaterials and devices. The analysis of surface interface properties mainly includes surface composition, surface morphology, surface structure, and surface energy state. The main characterization methods used in the experiment and the feedback information are shown in Table 1 [27,28,29,30,31]. Through these characterization methods, we can have a deep and comprehensive understanding of the surface or interface properties of nanomaterials and devices, which provide beneficial help for the further utilization of the surface/interface properties. Figure 2 contains three-dimensional images of the surface/interface topography using the confocal microscope with far-field configuration.

## 3. Influence of Surface and Interface on LED and Optimization Method

In the last few years, tremendous progress has been achieved in increasing the efficiencies, stabilities, and lifetime of light-emitting devices [32]. In this section, the application of nanostructured materials in LED devices will be introduced according to the dimension of materials, especially the influence of surface/interface properties on materials and devices, and the optimization mechanism will be summarized.

### 3.1. Influence of Surface and Interface of 0D Nanomaterials on LED and Optimization Method

Quantum dot-based LEDs (QD-LEDs) have attracted considerable attention owing to their high color purity, thermal stability, and size-dependent emission wavelength tenability, making them suitable candidates for next-generation solid-state lighting [13]. As noted in the introduction, QDs have a very large surface-to-volume ratio due to their small size. The surface/interface composition and structure of QDs can significantly affect the photoluminescence (PL) emission, charge injection, and charge transport. Thus, the surface/interface engineering of QDs plays a vital role in the realization of high performance QD-LEDs. In order to reduce the surface/interface effects and improve the device performance, some advances related to surface purification, different functional layers, the design of nanostructures, and different device architects have been made in the engineering of nanostructures and surfaces of QDs. The zero-dimensional material name, material structure, and optical properties of LED devices are summarized in Table 2.

#### 3.1.1. Surface Purification Methods

Inevitably, the synthesis of QDs will introduce many different type impurities, including metal carboxylate precursors, free ligands, and non-crystalline side products, which significantly diminish the stability, radiation efficiency, and potential applications of QDs in the field of nano-optoelectronic devices. Therefore, a surface purification for QDs is necessary for the improvement of the optical properties of QDs and device performance. Yang et al. fabricated the colloidal QDs from solution by the addition of a non-solvent [33]. The most common solvent and non-solvent pair was toluene and methanol, although other combinations including trichloromethane or hexanes as the solvent and methanol or acetone as the non-solvent have often been used as purification processes, as reported previously [34]. In addition, the hexane–methanol extraction process can also be used to purify the surface of QDs [35]. However, the non-solvent and hexane–methanol extraction approaches did not completely remove raw materials from the solution of colloidal QDs. Yang et al. developed a new purification scheme in which trichloromethane was selected as the solvent additive to enhance the solubility of the cadmium sources, while acetonitrile was selected as the solvent additive to precipitate the QDs, which was more effective than methanol [36]. Therefore, the improved purification scheme can effectively remove residual impurities in colloidal QD solution, including metal carboxylic acid precursors, non-volatile solvents, and non-crystalline by-products. Therefore, this benign purification process provides a good foundation for the surface engineering of quantum dots, and provides more opportunities for the photoelectric application of quantum dots. To solve the lifetime issue, Cao et al. tackle the hole barrier issue directly by tailoring the band-energy levels of QDs to reduce the injection barriers and improve device performance. A high-quality colloidal QD usually comprises an inorganic semiconductor core and a semiconductor shell with a wider energy bandgap to passivate dangling bonds of core surface and confine electron and hole wavefunctions for good luminescent properties and reliability [37].

#### 3.1.2. QDs Emitting Layer Method

The QDs emitting layer method, especially involving perovskite QDs, has significant potential and has been extensively studied for light-emitting devices. Organo-inorganic lead halide perovskite is a direct bandgap semiconductor material with many excellent properties and is attractive in electroluminescence (EL) applications [38,39,40,41,42]. Many high-efficiency perovskite light-emitting diodes, such as the green LED with peak external quantum efficiency (EQE) ≈ 8% [43] and the near-infrared LED with peak EQE of 11.7% [41], have the nanolevel structure of perovskite materials as their photon emission core. Kovalenko et al. synthesized monodisperse CsPbX_3_ perovskite quantum dots (X=Cl, Br and I, or Cl/Br and Br/I in the mixed halide system) [44,45]. These all-inorganic perovskite quantum dots cover the entire visible region with very high luminescence stability and photoluminescence quantum yields (PLQYs), and the gamut covers 140% of the NTSC standard. In addition, compared with organic–inorganic hybrid perovskite materials, all-inorganic perovskite QDs have better environmental stability [40,46,47].

#### 3.1.3. Surface Ligand Method

**Figure 3:**
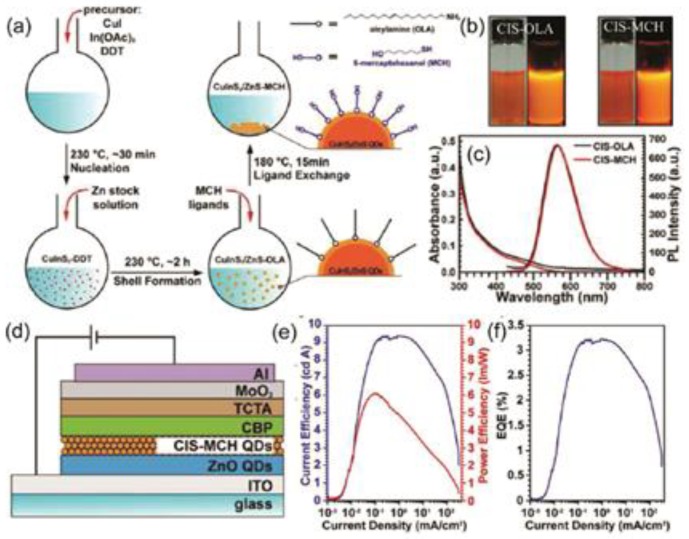
Schematic diagram of the in situ ligand exchange process and the relevant material and device measurement results. (**a**) The ligand exchange process and surface structure of the QDs. (**b**) The photos of QDs in solution before and after the ligand exchange process. (**c**) Absorption and photoluminescence (PL) results. (**d**) The device structure diagram of a quantum dot-based light-emitting diodes (QD-LED). (**e**,**f**) The power efficiency and current efficiency as a function of the current density. Reproduced with permission from reference [49].

The developments of surface coordination chemistry allow facile ligand-displacement reactions which enable the rational design of surface ligands for QDs used in LEDs [48]. Shen et al. report high-efficiency blue-violet QD-LEDs by using high quantum yield ZnCdS/ZnS graded core–shell QDs with proper surface ligands. Such ligand exchange results in an even greater increase in hole injection into the QD layer, thus improving the overall charge balance in the LEDs and yielding a 70% increase in quantum efficiency [11]. Next year, Zhong et al. developed an in situ ion exchange method to improve the performance of QD-LED devices [49]. The in situ ligand exchange process is shown in Figure 3. The results show that this method is very effective and the photoluminescence (PL) quantum yields are almost unchanged after the ligand exchange process. As a result, significant device performance improvements have been shown. Li et al. demonstrate a highly efficient solution-processed CsPbBr_3_ QD-LED through balancing surface passivation and carrier injection via ligand density control [50]. Compared to surface ligand exchange, the control of ligand density on QD surfaces is a more proper strategy to promote the performance of CsPbX_3_ QLEDs. In general, the solubility of QDs will decrease with the decrease of ligand size. On the other hand, the spatial separation between quantum dots caused by surface ligands will affect the colloidal stability of quantum dot solution [51]. In 2016, Peng et al. used “entropy ligands” [52,53] to improve the performance of nanocrystalline optoelectronic devices. Pan et al. have shown that the charge transfer characteristics of QD films can be regulated by using specially designed polymer ligands with colloids and perovskite groups instead of insulating ligands [54,55]. Therefore, a QD/polymer hybrid can be used as an important candidate material for the emitting layer of QLEDs [56]. Brown et al. demonstrated that the ligand-induced generation of surface dipoles is an effective way to control the absolute energy levels of QD films [57]. It is found that the strength of the surface dipole induced by the ligands can be regulated by the chemical binding group and dipole moment of the ligands, and the size of the surface energy level can be controlled. This method enables fine-tailoring of the band-energy calibration of PbS quantum dots, thus improving the optical performance of the device [58]. Yang et al. used this method to make QLEDs. Combined with the size control of QDs, the bandgap and band position of a PbS QD film can be fine-tuned by the quantum effect, so that the QD film can be used as electron transport layers (ETLs), hole transporting layers(HTLs), and the transmitting layer of LEDs [59].

#### 3.1.4. Core/Shell Interface Structure Method

Although surface purification is an important way to reduce impurities on the surface of QDs, the purification process may also bring about electron lone pairs or vacancies, which serve as surface traps by capturing excitons, thereby leading to non-radiative recombination. QDs often suffer from surface-related trap states, which act as non-radiative de-excitation channels for photogenerated charge carriers, thus decreasing their PL QDs. The photochemical stability, thermal stability, and photochemical stability of the quantum dot can be improved by covering the epitaxial layer and optimizing the growth parameters of the epitaxial layer, and the non-radiative recombination can be effectively reduced. Therefore, core–shell structure QDs are widely used to improve the performance of QLEDs. Bawendi et al. obtained high-quality CdSe/CdS core/shell QDs by using octanethiol and cadmium oleate as precursors and maintaining an appropriate growth rate at 310 °C. The obtained core/shell structure of the QDs has high uniformity; PLQYs can reach 97%, and the full width at half maximum (FWHM) of the PL peak is only 67.1 meV [60]. Pal et al. studied the optical properties of CdSe/CdS core/shell quantum dot films with different shell thicknesses and compared them with the luminescence properties of corresponding quantum dot solutions. The results show that the luminescence properties of core/shell materials are obviously improved, and the redshift effect of the PL spectrum is gradually weakened with the increase of the thickness of the CdS shell [61]. Li et al. showed that ZnCdSe-based core/shell QDs featuring a ZnS shell with 10 monolayers offered the highest external quantum efficiency of ~17%, which could compare favorably with the highest efficiency of green QLEDs with traditional multilayered structures [62]. The epitaxy of a gradient alloy shell on a core crystal can synthesize quantum dots with gradient band structures. Klimov et al. have shown that “smoothing” the shape of the constraint potential through the interfacial alloying of the core–shell interface can effectively inhibit an auger recombination in CdSe/CdS QDs [63,64]. Peng et al. prepared a series of small, scintillation-free and bleach-resistant high-quality CdSe/Cd*_x_*Zn_1−*x*_S and CdSe and CdSe*_y_*S_1−*y*_/Cd*_x_*Zn_1−*x*_S core/shell QDs. Quantum dots with a continuously adjustable luminous range and high PLQYs have important potential applications in LEDs [65].

#### 3.1.5. Optimization of LED Device Interface Architecture Methods

Device architecture using different charge transport layers (CTLs) and interfacial engineering is another important method to improve the performance of luminescent devices. In a typical QD-LED, apart from the QD-emitting layer, the charge-injection layers (CILs) and CTLs also significantly contribute to the overall device performance. The design of these layers can be made to favor the balance of the carrier injection, charge transport, and radiative recombination of excitons in the QD-emitting layer. Therefore, interfacial engineering between the QD-emitting layer and the CTL plays a critical role in enhancing the device performance of the QD-LEDs. Kim et al. used an all-solution processed method to fabricate highly efficient green QD-LEDs with an inverted architecture [66]. An interfacial polymeric surface modifier of polyethylenimine ethoxylated (PEIE) is inserted between a QD-emitting layer and a hole transport layer. At the same time, a MoO_x_ hole injection layer is solution deposited on top of the hole transport layer. Among the inverted QLEDs with varied PEIE thicknesses, the device with an optimal PEIE thickness of 15.5 nm shows record maximum efficiency values of 65.3 cd/A in current efficiency and 15.6% in external quantum efficiency (EQE). The all-solution processed fabrication of inverted QLEDs is further implemented on a flexible platform by developing a high-performing transparent conducting composite film of ZnO nanoparticles overcoated on Ag nanowires. The resulting flexible inverted device possesses 35.1 cd/A in current efficiency and 8.4% in EQE, which are also the highest efficiency values ever reported in flexible QLEDs. Apart from engineering the compositions, the size, structure, and shape control of QDs may provide additional benefits regarding the accessibility in band structure engineering and enhance the out-coupling efficiency in QD-LEDs. Nam et al. synthesized double-heterojunction nanorods consisting of two offset and staggered bandgaps, which offered independent control over the electron- and hole-injection processes in devices [12]. The out-coupling efficiency was significantly enhanced due to the nanorods and was assembled parallel to the substrates. More importantly, the anisotropic shape introduces a transition dipole along the rod axis. Liu et al. used the balanced charge-injection process to enhance the external quantum efficiency of nonblinking blue QD-LEDs [67]. Using nonblinking ZnCdSe/ZnS/ZnS QDs as the emissive layer, highly efficient blue QD-LEDs were prepared. The charge-injection balance within the QD active layer was improved by introducing a nonconductive layer of poly-(methyl methacrylate) (PMMA) between the electron transport layer (ETL) and the QD layer, where the PMMA layer takes the role of coordinator to impede excessive electron flux. The optimized LED device shows excellent performance such as a maximum luminance of 14,100 cd/m^2^, current efficiency of 11.8 cd/A, and external quantum efficiency (EQE) of 16.2%.

### 3.2. Influence of Surface and Interface of 1D Nanomaterials on LED and Optimization Method

1D nanomaterials possessing natural structures that can act as resonant cavities are ideal platforms to realize laser diodes and light-emitting diodes. Various nanostructures such as nanowires, nanotubes, and nanobelts have been synthesized to fabricate optoelectronic devices. In order to reduce the surface/interface effects and improve the device performance, some advances such as surface or interface structure design, interface control, and interface modification and modulation have been made in the engineering of 1D nanomaterial. The one-dimensional material name, material structure, and optical properties of LED devices are summarized in Table 3.

#### 3.2.1. Surface or Interface Structure Design Methods

**Figure 4:**
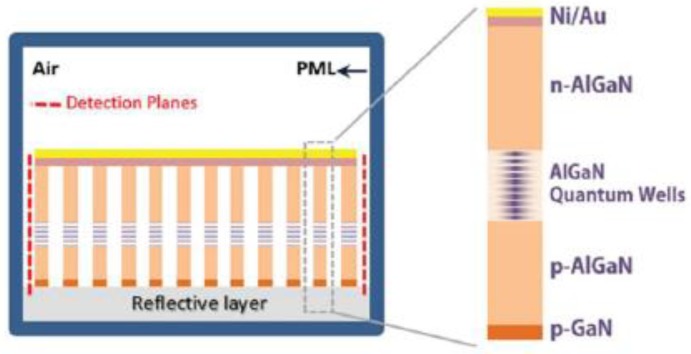
Schematic illustration of P-GaN/P-AlGaN/AlGaN core–shell UV LED structure. [71].

Similar to 0D nanomaterials, material and device structure design is a common method to suppress the surface states of 1D nanomaterials. For example, in order to reduce the reflection and improve the transmission of light, nanostructure arrays have been developed as effective antireflective surfaces [68]. By controlling the surface wetting properties of a polydimethylsiloxane release template, Liu et al. was able to pattern a random AgNWs network with uniform conducting property [69]. Modifying the core–shell structure is another method to overcome the total internal reflection at the semiconductor and air interface, improve the escape probability of the light, and consequently increase the light extraction efficiency [70,71], as shown in Figure 4. Zhang, Yao, and Zheng’s team used the high directionality of waveguide mode transmission and the efficient energy transfer of localized surface plasmon (LSP) resonances to increase the spontaneous emission rate of LEDs, respectively [72,73,74].

#### 3.2.2. Interface Control 1D Nanomaterial Methods

ZnO has inspired considerable attention to develop ultraviolet (UV) LEDs and LDs due to its wide direct bandgap of 3.37 eV and high exciton binding energy up to 60 meV. However, in the process of emission of 1D ZnO nanometer materials, it is difficult to avoid an extra emission from interface states. Hence, the interface design and optimization are necessary to realize efficient UV EL. So, both a high quality active layer and good interface at the p-i-n junction are critical factors to realize pure UV LED. You et al. successfully prepared the vertically aligned ZnO NRs and showed high crystal and optical quality. Nanostructured LED arrays were constructed by directly bonding ZnO NRs onto on AlN-coated p-GaN wafer. This simple and feasible method can effectively suppress the interface defects induced by the buffer layer formation [75,76].

#### 3.2.3. Interface Modification and Modulation 1D Nanomaterial Methods

As we all know, total internal reflection (TIR) will occur at the epitaxial layers/substrate interface and substrate/air interface because of the large difference in refraction indices. The surface/interface modification and modulation are effective methods to avoid the TIR. Guo et.al reported AlGaN-based 282-nm LEDs grown on nanopatterned sapphire substrates (NPSS), exhibiting 98% better performance relative to those grown on flat sapphire substrates [77]. The AlN epitaxial lateral overgrowth on pattered substrates or templates can not only improve the crystal quality of the overgrown epitaxial layers but also form embedded air voids in the AlN layer. The effective refraction index around the interface is thereby between the AlN layer and the substrates. The internal quantum efficiency (IQE) enhancement is estimated to be 60%, so the light-extraction efficiency (LEE) enhancement would be more than 20%. Lee et al. fabricated DUV LEDs on NP-AlN/sapphire templates, with air surrounding the AlN nanorods. Light emitted from the multiple quantum wells can propagate vertically by passing through the embedded nanostructures, and thus the TIR is avoided [78,79].

#### 3.2.4. Core/Shell Structure Methods

**Figure 5:**
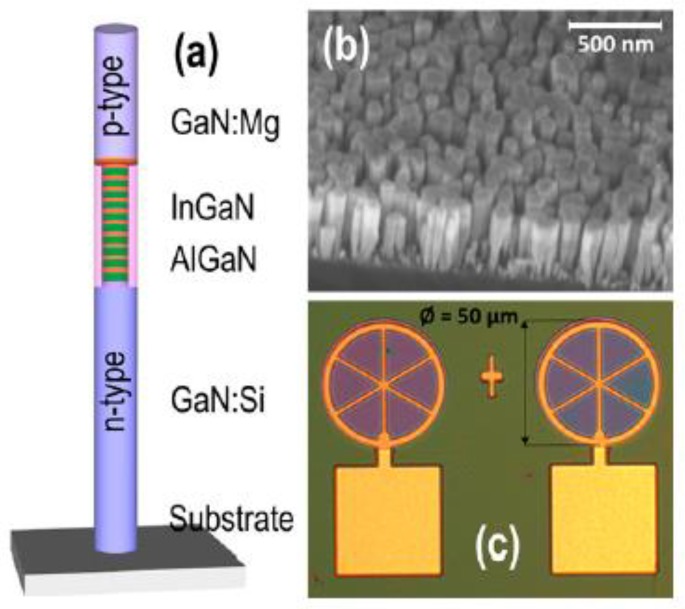
(**a**) Schematic diagram of nanowire LED with InGaN/AlGaN heterostructures. (**b**) The SEM image of InGaN/AlGaN nanowires. (**c**) The optical image and electrode pads of LEDs. Reproduced with permission from reference [89].

Due to the large surface-to-volume ratios of the 1D nanomaterials, the surface plays a key role in the optical properties of 1D nanometer material LEDs [80,81,82,83,84,85,86,87,88,89,90]. Due to the existence of surface states, a Fermi-level pinning effect will occur, and the resulting transverse electric field effect and related surface non-radiative recombination will be very unfavorable to one-dimensional nanostructured-led devices [80,81,82]. Optimizing the growth of core–shell structures can reduce non-radiative recombination and improve the photo-, electrical- and photochemical stability of 1D nanometer materials. Among them, InGaN/GaN and InGaN/AlGaN core–shell nanowires have attracted extensive attention due to their important applications in variable wavelength and UV LED devices. Ledig et al. characterized in detail the structural, optical, and electrical properties of InGaN/GaN core–shell structure LEDs [83]. It turns out that the 3D core shell structure design of the LED based on GaN has many advantages over conventional planar LED counterparts. In addition, in 2016, Müller et al. prepared the InGaN/GaN core–shell nanowires using the selective area metal organic vapor phase epitaxy method and studied the effects of InGaN thickness on cathodoluminescence spectroscopy [84]. The following year, InGaN/AlGaN and AlGaN core–shell tunnel junction nanowire LEDs were prepared by Philip’s research group and Sadaf’s research group, respectively [85,86]. The results show that the GaN-based nanowires LED can realize relatively high internal quantum efficiency from the deep green to red wavelength range. In 2018, Sim et al. designed highly efficient white LEDs using the 3D InGaN/GaN structure [87]. It can be proved by the experimental results that white LEDs based on dodecagonal ring structures are a platform enabling a high-efficiency warm white light-emitting source. Recently, high-performance InGaN/GaN and InGaN/AlGaN nanowire heterostructure LEDs were prepared by different research groups [88,89,90,91,92,93,94]. A schematic diagram of a nanowire LED with an InGaN/AlGaN core–shell heterostructure is shown in Figure 5. The results show that by controlling and optimizing the core shell structure, various surface defects and surface states can be effectively reduced and suppressed, and a high-quality crystal structure can be achieved, which can significantly improve the performance of LED devices.

### 3.3. Influence of Surface and Interface of 2D Nanomaterials on LED and Optimization Method

The application of 2D materials in LEDs mainly involves the synthesis of various thin films and the preparation of devices with heterogeneous LED structures [95,96,97,98]. Similar to zero and one-dimensional nanomaterials, it is also very important to quantify the influence of the 2D surface/interface on LED devices and to adopt effective methods to reduce the useless surface/interface recombination mechanism [99].

#### 3.3.1. Surface Modification and Interface Engineering Method

**Figure 6:**
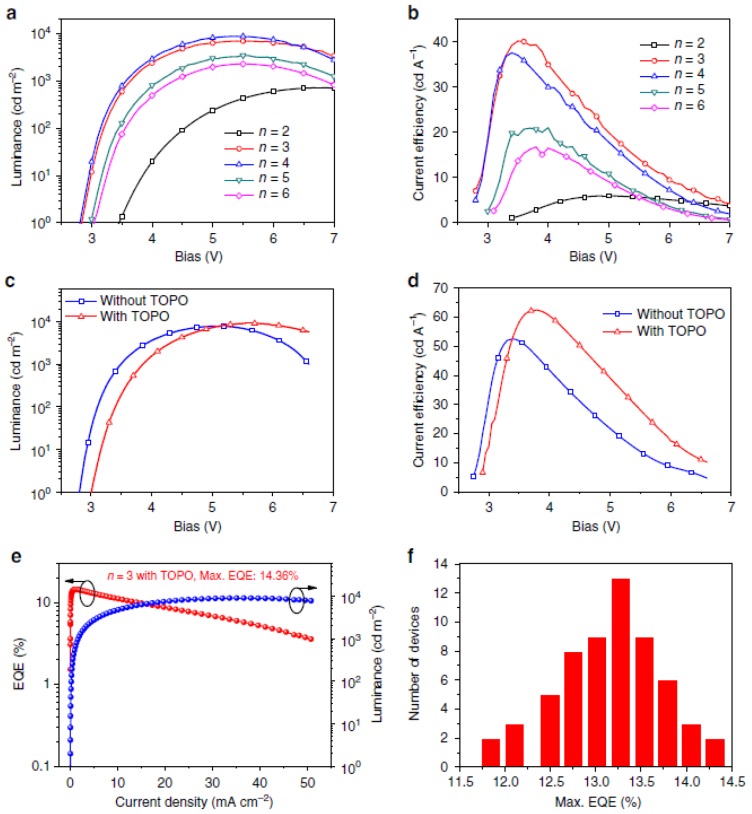
Device performance of perovskite LEDs with different compositions and surface passivation. (**a**) Luminance-voltage (L-V) curves with different compositions. (**b**) Current efficiency voltage (CE-V) curves with different compositions. (**c**) L-V curves with and without trioctylphosphine oxide (TOPO) layer. (**d**) CE-V curves without TOPO layer. (**e**) EQE with TOPO layer. (**f**) Maximum EQEs measured from 60 devices. Reproduced with permission from reference [104].

Carbon nanotubes and graphene are used for surface/interface modification or as the protecting layer to optimize LED device performance [100,101,102]. Due to the high refractive index, the light extraction efficiency of GaAs-based LED devices is limited. Nanoscale surface modification is an effective method to enhance the output light power of GaAs-based LED devices. Jin et al. report a simple method for nanostructure fabrication using super-aligned multiwalled carbon nanotube (SACNT) thin films as etching masks for top–down etching processes [100]. The morphology of the carbon nanotube (CNT) networks can be transferred to the substrate GaAs material at the macro scale. With this method, the nanostructured SACNT network morphology significantly increases the optical output power of GaAs devices in comparison with planar GaAs LED devices. Graphene is a useful material for conducting electrodes in LED device applications [101,102]. Seo et al. study the impact of the graphene quality on the performance of a hybrid electrode of graphene on AgNWs in GaN-based UV LED. The hybrid electrode using two-step graphene showed good ambient stability with stable sheet resistance over time. The UV LED using this TCE offered a low forward voltage, an increase in the EL intensity, and a reduction of efficiency droop. In addition, surface or interface passivation and the modulation of LED devices based on amine and perovskite materials have been studied [103,104,105,106]. Yang et al. made a green LED based on a quasi-2D perovskite composition and phase with surface passivation [104]. The surface passivation is realized through coating molecules of trioctylphosphine oxide on the surface of the perovskite thin film. The measured results of optimized LED based on quasi-2D perovskite reach a current efficiency of 62.4 cd A^−1^ and an external quantum efficiency of 14.36%, as shown in Figure 6. All the results show that the surface-sensitive characterization is expected to help to reveal the role that the interface plays in various devices and to identify specific strategies to regulate and tune the properties of the surface/interface, leading to enhanced device performance.

#### 3.3.2. Interface Structure Design Methods

**Figure 7:**
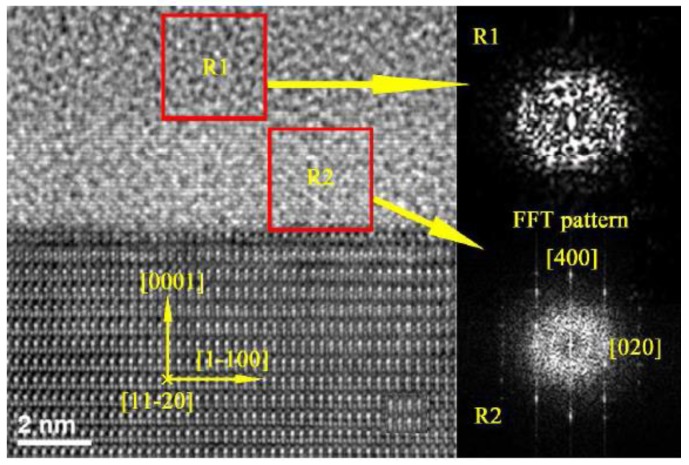
The high-resolution transmission electron microscope images across the SiN*_x_*/GaN interface. Reproduced with permission from reference [109].

For the 2D materials, core–shell, 3D pixel configuration, back-end-of-line material, and device structures are designed to optimize the device performance [107,108,109,110,111,112,113]. For example, Zhang et al. insert a 4-nm Si_3_N_4_ layer between the ZnSe core and the CdS shell of p-ZnSe/n-CdS core–shell heterojunctions to passivate the interface defects and reduce the recombination and the saturation current [107]. Liu et al. study the band states at the crystallized interface between GaN and SiNx and the influence of interface roughness on the material, as shown in Figure 7 [109]. Zhang et al. designed a three-dimensional reflective concave structure coated with a high refractive index material and achieved an increase in OLED display pixels by embedding the OLED into the three-dimensional reflective concave structure. This structure allows the coupling region of the light to be defined so that as much of the coupling interior is emitted to the filled region and then redirected. The optical simulation results show that if the optimized structure and highly transparent top electrode material are adopted, the efficiency of light extraction can be improved by ≈ 80%. [110]. Bulling and Venter use two methods to improve light extraction efficiency: first, the design of an improved back-end-of-line (BEOL) light directing structure, and second, the use of surface texturing. The design of an optimized pipe-like BEOL light directing structure has resulted in a 1.35-factor improvement in luminance and a 1.38-factor improvement in light extraction efficiency over the previously designed parabolic BEOL light directing structure; furthermore, it has also resulted in an improved BEOL light-directing structure for improved light extraction efficiency. In addition, the directionality of the light emission radiation pattern has also significantly improved. Once the internal TIR is reduced and the light radiation propagation is improved, surface texturing techniques can be used to further improve the light extraction efficiency [108]. Recently, Lei et al. used the surface texture and LSP coupling effect to enhance the light extraction efficiency for InGaN/GaN LEDs [113].

## 4. Conclusions and Perspective

In summary, we introduce the effects of surface/interface properties of nanostructured materials on light-emitting diodes, taking some II–VI, III–V, IV, and perovskite nanomaterials as representatives. According to the review, we can see that the surface/interface properties of nanostructured materials are an important factor affecting the performance of the device, which involves the generation of carriers, recombination, separation, collection, and other dynamic processes. Most important of all, as shown in the paper, through surface or interface engineering such as surface purification, surface ligands, or introducing the core/shell structure and others in constructing optoelectric devices, the performance could be improved remarkably. Therefore, continuing to study the surface/interface of nanostructured light-emitting devices from the microscopic perspective and understand the correlation between the properties of nanostructured devices in depth is crucial. Even though this progress in the improvement of the device performance of LEDs is very encouraging, there are several shortcomings and challenges that stand in the way of commercialization. The following aspects are critical to improving the device performance and accelerating the commercialization of LEDs.

1. Reducing Surface/Interface Recombination

As we all know, when the atomic lattice is abruptly broken at a surface/interface, unsatisfied dangling bonds (or foreign bonds) introduce electronic energy levels inside of the bandgap that enhance electron–hole non-radiative recombination at the surface/interface by acting as stepping stones for charge carrier transitions between the conduction and valence bands. However, achieving these values in operational device architecture has remained elusive because contacting the nanomaterials with extracting contacts generally induces new, non-radiative loss pathways at the surface, resulting in a decrease in the PLQE and PL lifetime.

2. The Choice of the Appropriate Surface Ligands

The surface ligand methods play an important role in improving the properties of nanomaterials and devices. On the one hand, surface ligands can effectively combine with surface atoms to passivate surface defects and reduce the surface states of materials. On the other hand, the intrinsic insulation of the surface ligands will affect the effective charge injection and transmission characteristics of the emitting layer, and reduce the efficiency and performance of the nanoluminescent devices. Therefore, balanced exciton recombination and charge injection/transport are key to the effective use of the method of surface ligand in nanostructured LEDs.

3. In-Depth Understanding of the Interactions of Nanomaterials with CTLs

It is generally considered that the exciton quenching of nanomaterials is critical to the performance of nanostructure LEDs. In nanostructure LEDs, nanomaterials can acquire a net charge due to the interactions with the CTLs, and the excess charges in the charging nanomaterials can lead to exciton quenching by non-radiative energy transfer to the CTLs or the defects within the CTLs films, thus diminishing the device efficiency. Therefore, it is necessary to develop effective characterization techniques to study the interactions of the nanomaterials with different CTLs in order to gain a more comprehensive understanding of the mechanisms.

4. Reduce Internal/Reflection TIR Effect

As mentioned earlier, TIR occurs at the epitaxial layers/substrate interface and substrate/air interface because of the large difference in refraction indices. A large amount of photons are trapped inside the LED structure and finally absorbed after multiple internal reflections. Disturbing the TIR at the interfaces would be beneficial to achieve high-efficient LEDs. Therefore, how to use highly reflective techniques and surface/interface modification to mitigate TIR’s influence on nanostructured light-emitting devices is still a difficult task.

## Figures and Tables

**Table 1:** Surface/interface characterization of nanomaterials and devices ^1^.

Analysis Method	Acquired Information
Auger electron spectrum (AES) X-ray photoelecgon spectroscopy (XPS) Secondary ion mass spectrometry (SIMS) Ion scattering spectroscopy (ISS)	Surface/interface composition
Transmission electron microscopy (TEM) Scanning electron microscope (SEM) Scanning tunneling microscopy (STM) Atomic force microscope (AFM)	Surface/interface morphology
Low-energy electron diffraction (LEED) Field ion microscope (FIM) High-resolution transmission electron Microscopy (HRTEM)	Surface/interface structure
X-ray photoelecgon spectroscopy (XPS) Ultraviolet photoelectron spectroscopy (UPS) Electron energy loss spectroscopy (EELS) Ion neutralization spectroscopy (INS) Deep level transient spectrum (DLTS) Pulsed I–V measurement Conductance	Surface/interface energy state

^1^ References [27,28,29,30,31].

**Table 2:** The summaries of zero-dimensional material name, material structure, and optical properties of light-emitting diode (LED) devices. QD: quantum dots.

Materials	Structures	Spectral Range of LEDs	Ref.
CsPbBr_3_	Nanocrystals	Green	[34]
CdSe/ZnS	QDs	Blue, Green, Red	[36]
CdSe/Cd_1-*x*_Zn*_x_*Se	QDs	Red	[37]
CsPbBr_3_	QDs	Deep-red	[38]
CH_3_NH_3_Pb_3-*x*_Cl_x_	Films	Near-infrared	[39]
CsPbX_3_	QDs	Red, Orange, Green, Blue	[40]
NFPI_7_	QWs	Near-Infrared	[41]
CH_3_NH_3_PbI_3_	Quasi-2D	Near-Infrared	[42]
CH_3_NH_3_PbBr_3_	QDs to Nanograins	Green	[43]
CsPbX_3_ (X = Cl, Br, I)	Nanocrystals	Blue, Green	[44]
CsPbX_3_ (X = Cl, Br, I)	Nanocrystals	Blue to Red	[45]
CsPbBr_3_	QDs	Blue to Red	[46]
CsPbX_3_	QDs	Blue to Red	[47]
CuInS_2_	QDs	Green	[49]
CsPbX_3_	QDs	Green	[50]
CsPbI_3_	Nanocrystals	Red	[55]
Colloidal	QDs	Near-Infrared	[59]
CdSe/CdS	Nanocrystals	~	[60]
CdSe/CdS	QDs	Red	[61]

**Table 3:** The summaries of one-dimensional material name, material structure, and optical properties of LED devices.

Materials	Structures	Spectral Range of LEDs	Ref.
Ag	Nanowires	Green	[69]
InGaN/GaN	Nanostructure	~	[70]
AlGaN	Nanowires	Deep ultraviolet	[71]
ZnO/MgZnO	Nanorods	Ultraviolet	[72]
ZnO/GaN	Nanorods	Ultraviolet	[73]
CdS	Nanowires	Red	[74]
ZnO	Nanowires	Ultraviolet	[75]
AlGaN	Nanowires	Ultraviolet	[77]
AlGaN	Nanoscale	Deep ultraviolet	[78]
InGaN/GaN/AlGaN	Nanowires	White	[80]
GaN	Nanowires	White	[81]
InGaN/AlGaN	Nanowires	Full color	[82]
InGaN/GaN	Nanorods	Green	[84]
InGaN/AlGaN	Nanowires	Green, Yellow	[85]
AlGaN	Nanowires	Ultraviolet	[86]
InGaN/AlGaN	Nanowires	Full color	[89]
InGaN/AlGaN	Quantum disks	~	[90]
